# Deep-Learning-Based Arrhythmia Detection Using ECG Signals: A Comparative Study and Performance Evaluation

**DOI:** 10.3390/diagnostics13243605

**Published:** 2023-12-05

**Authors:** Nitish Katal, Saurav Gupta, Pankaj Verma, Bhisham Sharma

**Affiliations:** 1 School of Electronics Engineering, Vellore Institute of Technology, Chennai 600127, Tamil Nadu, India; nitishkatal@gmail.com; 2 University Centre for Research and Development, Academic Unit 2, Chandigarh University, Mohali 140413, Punjab, India; pankaj.e15048@cumail.in; 3 Chitkara University Institute of Engineering and Technology, Chitkara University, Rajpura 140401, Punjab, India

**Keywords:** arrhythmia detection, deep learning, ECG, healthcare, machine learning

## Abstract

Heart diseases is the world’s principal cause of death, and arrhythmia poses a serious risk to the health of the patient. Electrocardiogram (ECG) signals can be used to detect arrhythmia early and accurately, which is essential for immediate treatment and intervention. Deep learning approaches have played an important role in automatically identifying complicated patterns from ECG data, which can be further used to identify arrhythmia. In this paper, deep-learning-based methods for arrhythmia identification using ECG signals are thoroughly studied and their performances evaluated on the basis of accuracy, specificity, precision, and F1 score. We propose the development of a small CNN, and its performance is compared against pretrained models like GoogLeNet. The comparative study demonstrates the promising potential of deep-learning-based arrhythmia identification using ECG signals.

## 1. Introduction

Arrhythmias, irregular heart rhythms, are a major health concern, affecting millions globally. The status of arrhythmia can range from benign, with a minimal impact on health, to severe, which can be fatal by causing cardiac arrest, stroke, etc. Hence, its early detection can aid in effective clinical management [1]. An electrocardiogram (ECG) is the principal procedure employed for effective arrhythmia detection, but diagnosis can be challenging because of the subtle disposition of symptoms. Lately, advancements in deep-learning algorithms have offered a promising solution for automation and accurate diagnosis in medical applications [2]. 

The early detection of arrhythmias is crucial, as timely treatments, like medications and lifestyle changes, can be initiated, thus preventing further complications. Also, the detection of certain arrythmia types, like ventricular tachycardia or atrial fibrillation, is essential and can prevent the occurrence of severe events like cardiac arrest or stroke. This can aid doctors in instigating preventive procedures such as implantable cardioverter defibrillators (ICDs) or anticoagulant therapy, reducing the risk of life-threatening events. Furthermore, arrhythmia detection plays a vital role in personalized treatment [3,4]. Arrhythmias can have various underlying causes and may require different treatment approaches. The accurate detection and classification of arrhythmias using ECG signals can aid in identifying the specific type of arrhythmia and tailoring treatment plans accordingly. This personalized approach ensures that patients receive the most effective therapies.

In addition, the ability to remotely monitor arrhythmias has transformative potential in healthcare. Arrhythmias often occur sporadically or intermittently, making their detection challenging using traditional methods that rely on short-term ECG recordings. EEG signals, on the other hand, can be continuously monitored over extended periods, allowing for the detection of transient arrhythmias that may go unnoticed in standard clinical assessments [5]. Remote monitoring and telemedicine, facilitated by continuous EEG monitoring, enable patients to receive timely medical attention regardless of their physical location, enhancing access to healthcare services and improving patient care. Moreover, arrhythmia detection has implications for reducing healthcare costs. The timely detection and effective management of arrhythmias can help decrease healthcare costs associated with emergency department visits, hospitalizations, and long-term complications. By implementing automated arrhythmia detection systems, healthcare providers can optimize resource allocation, streamline patient care, and potentially alleviate the financial burden on individuals and healthcare systems [6].

Although traditional methods, such as ECG, have been the standard for arrhythmia detection, they possess certain limitations that motivate the exploration of alternative approaches. ECG recordings provide valuable information about heart activity, but they are primarily focused on measuring electrical signals directly from the heart. Additionally, ECG-based arrhythmia detection heavily relies on human interpretation, which can introduce subjectivity and variability in diagnoses. The accuracy of traditional methods is dependent on the expertise of the interpreting healthcare professional, and the misinterpretation or misclassification of arrhythmias can occur, potentially leading to incorrect treatment plans or missed opportunities for intervention. Moreover, traditional methods may struggle with the detection of certain types of arrhythmias that exhibit complex or atypical patterns. For instance, detecting arrhythmias during exercise or under specific physiological conditions may pose challenges due to the dynamic nature of heart rhythms. Traditional methods may not capture these variations adequately, potentially leading to missed diagnoses or delayed treatment.

These limitations of traditional arrhythmia detection methods highlight the need for alternative approaches that can enhance accuracy, objectivity, and efficiency in detecting and classifying arrhythmias. Thus, there is huge potential for applying deep learning for the accurate and automated recognition of arrythmias by ECG signals.

Deep learning, a subset of artificial intelligence, has demonstrated remarkable success in various fields, including computer vision, knowledge engineering, and medical image analysis [7]. Its ability to automatically learn multifaceted patterns and characteristics from raw information makes it suitable for examining ECG signals and detecting arrhythmias. Deep learning algorithms can capture intricate temporal and spatial relationships within the ECG signals, enabling the accurate classification of different arrhythmia types. The integration of deep learning algorithms with ECG signals offers several advantages for arrhythmia detection. Firstly, deep learning models can handle the inherent complexity and variability of ECG data, capturing the subtle patterns and features associated with different arrhythmias. These models can learn from large-scale datasets, encompassing diverse arrhythmia cases, to generalize well and improve detection accuracy.

Furthermore, deep learning models can provide automated and real-time arrhythmia detection, reducing reliance on human interpretation and increasing efficiency. Once trained, these models can analyze ECG signals in real time, rapidly identifying abnormal heart rhythms and alerting healthcare professionals for further assessment and intervention. This automation streamlines the detection process, enabling quicker diagnosis and timely treatment. Additionally, the integration of deep learning with ECG signals allows for continuous monitoring, capturing transient or intermittent arrhythmias that may go unnoticed in short-term ECG recordings. Continuous monitoring enhances the ability to detect and characterize arrhythmias, providing a comprehensive assessment of a patient’s heart rhythm over an extended period. This continuous monitoring capability, coupled with deep learning algorithms, facilitates remote monitoring and telemedicine applications, empowering patients and improving access to healthcare services.

The objective of the proposed work was to investigate the potential of deep learning techniques for accurate and automated arrhythmia detection using ECG signals as inputs. Specifically, the study aimed to develop and compare deep learning models for classifying arrhythmias using ECG data, ultimately evaluating their performance.

The paper will be structured as follows: The introduction highlights the significance of arrhythmia detection and the potential of ECG signals and deep learning for accurate and automated detection. This is followed by a review of previous studies in the literature. Additionally, the methodology section will describe the dataset used, the deep learning model architecture, and the evaluation metrics employed for assessing the performance. This is followed by the results and discussion sections, where the performance of the small CNN is contrasted with that of GoogLeNet. The findings exhibit that the proposed small CNN performed better than GoogLeNet in detecting arrhythmia using ECG signals. The small CNN model exhibited a high accuracy of 91.2%. This indicates that the presented approach has the capacity to be used as an effective means for automated arrhythmia detection. The discussion also highlights the advantages of the small CNN model, such as its simplicity and efficiency compared to the more complex GoogLeNet models.

## 2. Literature Review

The variation in heart activity can be captured by an ECG, a type of non-invasive technology. Through the placement of electrodes on the patient’s body, an ECG machine is able to monitor the heart’s rhythm [8]. ECG analysis is a challenging undertaking due to the variation in heartbeats, the tiny amplitude of the collected signal, and the difficulty of recognizing its components. The challenge here is to precisely identify and classify various types of arrhythmias [9]. The precise study of ECG reports requires a proficient doctor, which may lead to manual errors. These errors can be overcome by designing an intelligent automated system using the current attractive technology of machine learning and deep learning.

Although there are several methods in the literature for detecting arrhythmia, studies have concentrated more on noise filtering from ECG signals [10,11,12,13,14,15]. Various machine learning models have been put forth based on signal segmentation [16,17], manual feature extraction [18], and support vector machines (SVMs) [19,20]. Further, higher-order statistics and Hermite functions have been employed along with a variety of machine-learning-based methods to extract more features [21,22,23]. The above-mentioned methods have certain limitations, such as the considerable cost, the time required, and the manual pre-processing of the signals. To overcome these limitations, researchers have used deep learning models like CNNs [24,25] and recurrent neural networks (RNNs) [26,27] to study ECG signals, but their work still relies on the pre-processing of the signals, which can result in information loss.

Recently, deep learning has revealed an astonishing improvement in the domain of medical diagnosis. Some of the recent important literature relevant to this field has exploited this technology for automatic heart detection. DL-based ECG classification can more effectively characterize the nature of ECG signals when compared to conventional machine-learning-based classification methods like clustering and SVMs. This is possible because of the effective multi-level abstraction of the feature extraction capability [28]. Therefore, the classical neural-network-based methods and SVM classifiers have been superseded by DL or DNNs in arrhythmia classification [29]. In [30], a DNN with raw ECG data as the input signal was proposed. This did not require prior handcrafted feature extraction. The research in [24,25] demonstrated that DNNs perform better when some temporal variables are used along with raw data inputs. In the literature, many studies have used different datasets: some have used freely accessible ones like the MIT-BIH Arrhythmia Database [17,31] and the PhysioNet Challenge datasets [32], while others have collected and annotated their own data [33]. The authors of [34] used the MIT-BIH Arrhythmia Database, which was created using a Holter device to capture long-term ECG data. This database captures the infrequent events in the heart that may also lead to arrhythmia disease. The authors of [35] validated the proposed strategy by using three databases: the MIT-BIH Arrhythmia Database, INCART, and the SVDB. From the conclusions of these studies, it can be demonstrated that deep learning techniques hold potential in achieving the high-accuracy and early identification of arrhythmias. This could pave the way for more precise and effective clinical decision support systems in the area of arrhythmia diagnosis and therapy.

A comparative study of the methodologies used in the literature identifies a variety of deep learning methods for the detection of arrhythmias. CNNs have demonstrated remarkable performance in feature extraction from raw ECG signals [24,25]. RNNs excel at modeling temporal relationships, although they can be noise-sensitive and may need a lot of data per-processing step [26,27]. Hybrid architectures of deep learning [35,36] appear to include the advantages of both CNNs and RNNs.

## 3. Materials and Methods

Here, the various methodologies employed in the current study are discussed.

### 3.1. Dataset

The work considered the use of 3 PhysioNet databases, namely the MIT-BIH Arrhythmia Database, the MIT-BIH Normal Sinus Rhythm Database, and the BIDMC Congestive Heart Failure Database, to classify ECG samples of subjects with cardiac arrhythmia (ARR), normal sinus rhythm (NSR), and congestive heart failure (CHF), respectively. The MIT-BIH ARR Database contains 48 samples of 30 min excerpts from two-channel ECH recordings of 47 subjects. The MIT-BIH NSR Database contains 18 long-term ECG recordings for subjects with no arrythmia from the Arrhythmia Laboratory at Boston’s Beth Israel Hospital. The BIDMC CHF Database contains long-term ECG recordings from 15 subjects with severe congestive heart failure (NYHA class 3–4). In total, the study used 162 ECG recordings from these 3 PhysioNet databases, of which 96 ECG records belonged to subjects with ARR, 30 NSR, and 36 CHF. Table 1 provides an overview of the databases for arrhythmia research. This table presents the key attributes and details of three databases commonly applied in arrhythmia research, namely the MIT-BIH ARR Database, MIT-BIH NSR Database, and BIDMC CHF Database. The attributes covered include the type of database, data collection methods, number of subjects, recorded parameters, annotations, location, technical specifications, sampling rates, purpose, challenges, use cases, and relevant references.

### 3.2. Pre-Processing Steps Applied to ECG Signals

Pre-processing and signal transformation steps are applied to ECG signals in the databases to prepare them for training purposes. This paper explores the representation of ECG signals as scalograms. These scalogram images are obtained by applying the continuous wavelet transform to the ECG signals. This transformation allows for the extraction of time–frequency information from the signals, which can then be visualized as scalograms. These scalogram images provide a more comprehensive representation of the ECG signals, capturing both their temporal and spectral characteristics. Scalograms offer a better representation than the traditionally used spectrograms as they provide a more detailed and localized view of the signal’s frequency content over time. This can be particularly useful in analyzing ECG signals, as it allows for the identification of specific frequency components that may be indicative of certain cardiac conditions or abnormalities. Additionally, the use of scalograms can aid in the detection and classification of various signal artifacts or noise, enhancing the accuracy and reliability of ECG analysis.

The various pre-processing steps implemented to generate the scalograms were as follows:

1. Filtering the ECG signals to remove any unwanted noise or artifacts.
2. Applying a time–frequency analysis technique, such as the continuous wavelet transform, to obtain the scalogram representation.
3. Adjusting the parameters of the time–frequency analysis, such as the wavelet type and scale range, to optimize the visualization of specific frequency components.
4. Visualizing the scalogram to observe any patterns or abnormalities in the frequency content over time.
5. Using automated algorithms or artificial intelligence to analyze the scalogram and detect any abnormal ECG patterns, such as arrhythmias.

**Figure 1:**
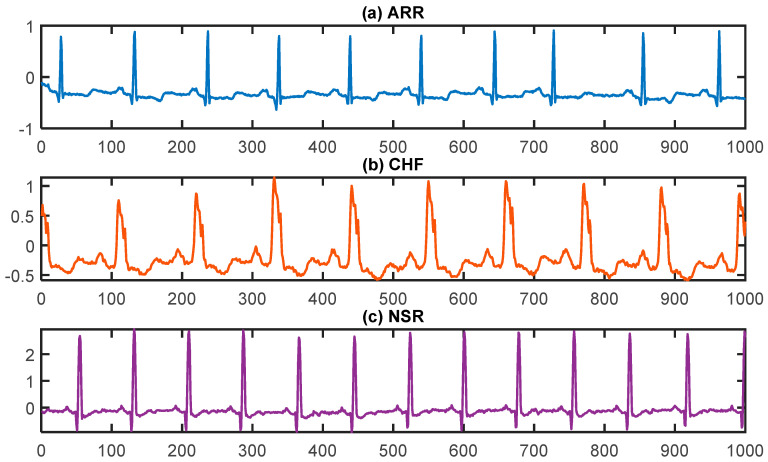
Plots of ECG signals for (**a**) cardiac arrhythmia (ARR), (**b**) congestive heart failure (CHF), and (**c**) normal sinus rhythm (NSR), respectively.

The visualization of ECG signals for ARR, NSR, and CHF is presented in Figure 1. In Figure 1, it can be observed that all three ECG patterns have their own characteristic curve shapes. The ARR pattern exhibits irregular and abnormal waveforms, indicating an irregular heart rhythm. In contrast, the NSR pattern shows a regular and consistent waveform, indicating a healthy heart rhythm. Lastly, the CHF pattern displays a distorted waveform with varying amplitudes, representing the compromised function of the heart due to fluid accumulation. These distinct curves enable medical professionals to accurately diagnose and differentiate between different cardiac conditions by analyzing the ECG signals. By analyzing the irregular and abnormal waveforms, medical professionals can identify conditions such as atrial fibrillation or ventricular tachycardia, which require specific treatment approaches. On the other hand, the regular and consistent waveform of NSR helps confirm a healthy heart rhythm and rule out any significant cardiac abnormalities. The distorted waveform and varying amplitudes of the CHF pattern provide valuable insights into the compromised function of the heart, allowing doctors to prescribe appropriate medications and interventions to manage fluid accumulation and improve heart function. Overall, the analysis of ECG signals plays a crucial role in accurate diagnosis and effective treatment planning for patients with cardiac conditions.

**Figure 2:**
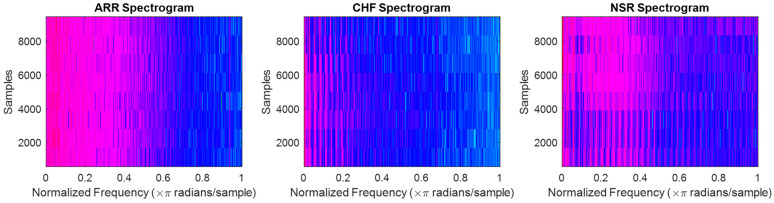
Spectrograms of the ECG signals for cardiac arrhythmia (ARR), congestive heart failure (CHF), and normal sinus rhythm (NSR), respectively.

The spectrograms for ARR are shown in Figure 2a, which displays the frequency content of the heart’s electrical activity over time. The spectrograms reveal important information about the irregularities in the heart rhythm, such as the presence of atrial fibrillation or other arrhythmias. By analyzing spectrograms, doctors can assess the severity of ARR and tailor treatments accordingly. The spectrograms for CHF are shown in Figure 2b, and it can be observed that there are significant differences in the frequency content compared to the spectrograms of ARR. This indicates that the electrical activity of the heart in patients with CHF is distinct from that of patients with ARR. Analyzing the spectrograms for CHF can help doctors identify specific patterns and abnormalities associated with this condition, enabling them to provide targeted treatments and interventions. Finally, the spectrograms for NSR are shown in Figure 2c and highlight the normal electrical activity of the heart. The spectrograms show consistent and regular patterns, with a clear separation of frequency bands. This confirms that patients with NSR have a healthy and properly functioning heart. By comparing the spectrograms for CHF, ARR, and NSR, doctors can gain valuable perspectives on the electrical operation of the heart and make informed decisions regarding patient care and treatment options.

**Figure 3:**
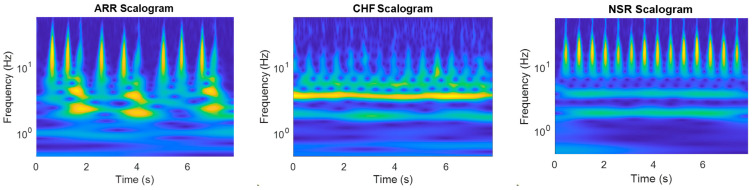
Scalograms of the ECG signals for cardiac arrhythmia (ARR), congestive heart failure (CHF), and normal sinus rhythm (NSR), respectively.

In Figure 3, the scalograms of all three signals are shown, and it can be observed that the scalograms for CHF and ARR show irregular and fragmented patterns, with overlapping frequency bands. This indicates abnormal electrical activity in the heart, which can be indicative of heart failure or arrhythmia. These findings can help doctors identify and diagnose these conditions, allowing for appropriate interventions and management strategies to be implemented. Furthermore, the comparison of scalograms assists in monitoring the effectiveness of treatment and assessing the progression of the disease over time. Thus, scalograms offer a better representation of ECG signals than time-series signals and spectrograms because they provide a clear visualization of the changes in heart activity. This allows medical professionals to accurately analyze and interpret the data, leading to more accurate diagnoses and treatment plans. Additionally, the use of scalograms can help identify any abnormalities or irregularities in the ECG signals that may not be easily visible in other types of representations.

### 3.3. Deep Learning Architecture

This paper considers the following deep learning architectures:

*A*. ***GoogLeNet:*** This is a deep learning architecture that introduced the concept of inception modules, which are able to capture information at different scales and provide a better representation of the input image. It also makes use of 1 × 1 convolutions to lessen the number of parameters and has a relatively low computational cost compared to other architectures.
*B*. ***Custom CNN:*** This work also considers the development of a small CNN. This network consists of a total of 21 layers, comprising a sequence of a 2D convolution layer followed by a batch normalization layer, a ReLu layer, and finally a 2D max pooling layer; this sequence of layers is repeated 4 times and has a filter size of 16, with 4, 8, and 16 filters, respectively. The custom CNN model considered in this study has approximately one fourth the number of layers of GoogLeNet, which has 144 layers.

### 3.4. Training Procedure

**Figure 4:**
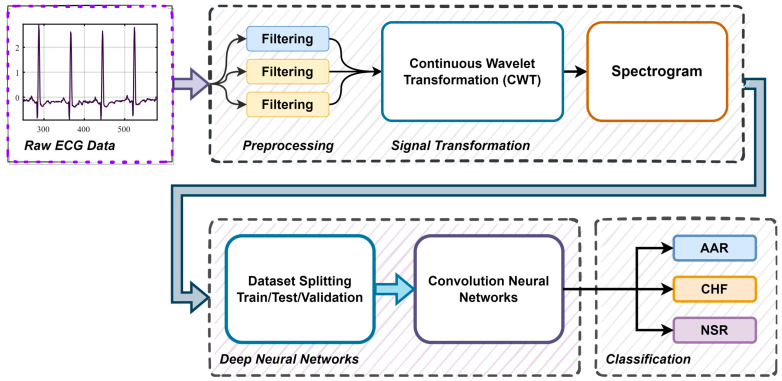
A block diagram representation of the arrythmia detection process using ECG signals.

The dataset encompassed the three PhysioNet databases for classifying the ECG samples as subjects with ARR, NSR, and CHF. Its development was carried out using MATLAB. Figure 4, shows the block diagram representation of the arrythmia detection process using ECG signals. The various steps involved in the training procedure were as follows:

*A*. *Pre-processing and Signal Transformation*: We pre-processed the data to remove noise and artifacts and segmented them into individual heartbeats or time windows. We converted the ECG signals to scalograms using CWT and employed the obtained scalograms to train the model.
*B*. *Data Splitting*: We divided the dataset into training, validation, and testing sets in a ratio of 70% training, 15% validation, and 15% testing. The work used the 10-fold cross-validation method.
*C*. *CNN Model Selection*: In the proposed work, a small CNN network was designed for the automated detection of ARR, CHF, and NSR. The considered SmallNet had approximately one fourth the number of layers of GoogLeNet, which has 144 layers. To establish the efficacy of the proposed model, it was compared with a pre-trained GoogLeNet CNN model.
*D*. *Definition of Loss Function and Optimizer*: We defined a suitable loss function and an optimizer (e.g., Adam, SGDM) for the classification task.
*E*. *Training*: We passed the training data through the modified CNN model. We calculated the loss between the predicted outputs and the actual labels. We backpropagated the gradients and updated the weights of the unfrozen layers using the chosen optimizer. We monitored training and validation loss and applied early stopping as needed.
*F*. *Model Evaluation*: We evaluated the fine-tuned model on the held-out test set to assess its performance using metrics such as accuracy, ROC, AUC, sensitivity, specificity, precision, and F1 score.

### 3.5. Performance Evaluation

Deep learning models are evaluated using metrics such as the confusion matrix, sensitivity, specificity, ROC curve, AUC, precision, and F1 score. The confusion matrix provides a comprehensive overview of the model’s classification accuracy, while the ROC curve illustrates the trade-off between true-positive and false-positive rates. The AUC, derived from the ROC curve, quantifies the model’s overall performance, with a higher AUC indicating better classification. The ROC curve is a powerful tool for visual assessment, allowing for the selection of the optimal threshold for sensitivity and specificity. The AUC simplifies the evaluation process and comparisons between models or approaches. The details of each performance metric considered in this study are given below:

*A*. *Confusion Matrix*

The confusion matrix is a tabular representation of a model’s classification performance. It consists of four essential components:

- True positives (TPs): instances where the model correctly predicted positive cases.
- True negatives (TNs): instances where the model correctly predicted negative cases.
- False positives (FPs): instances where the model incorrectly predicted positive cases when they were actually negative.
- False negatives (FNs): instances where the model incorrectly predicted negative cases when they were actually positive.

By examining these components, we gain insight into how well the model distinguishes between positive and negative cases, which is especially critical in applications like medical diagnosis.

*B*. *ROC Curve*

The ROC curve is a graphical representation of a model’s performance across various classification thresholds. It plots the true-positive rate (sensitivity) against the false-positive rate (1-specificity) as the threshold for classification changes. This curve provides a visual depiction of the trade-off between correctly identifying positive cases and incorrectly classifying negative cases. A model with a steeper ROC curve generally has better discrimination ability. The ROC curve is particularly useful when deciding on an optimal threshold for decision making, as it allows one to select a threshold that aligns with one’s specific objectives.

*C*. *AUC*

The AUC is a scalar value derived from the ROC curve. It enumerates the inclusive performance of the model by measuring the area under the ROC curve. In the context of identifying heart arrhythmias or any classification task, a higher AUC indicates better performance in distinguishing between different classes. The AUC is a valuable metric for comparing different models or approaches, as it condenses complex performance data into a single number, simplifying the evaluation process.

*D*. *Sensitivity*

Sensitivity, also known as the true-positive rate or recall, measures the ability of a model to correctly identify positive cases. Sensitivity is crucial in medical diagnostics, especially when dealing with conditions like diseases. High sensitivity means fewer false negatives, ensuring that individuals with the condition are correctly identified.

$$Sensitivity=\frac{TP}{TP+FN}$$

*E*. *Specificity.*

Specificity measures the ability of a model to correctly identify negative cases. Specificity is vital to rule out individuals without a particular condition. High specificity means fewer false positives, ensuring that healthy individuals are correctly identified as such.

$$Specificity=\frac{TN}{TN+FP}$$

*F*. *Precession.*

Precision, also known as the positive predictive value, quantifies the accuracy of positive predictions made by a model. Precision is crucial when the focus is on the accuracy of positive predictions. In medical scenarios, high precision implies that when a test predicts a positive result, it is likely to be accurate.

$$Precision=\frac{TP}{TP+FP}$$

*G*. *F1 Score.*

The F1 score is the harmonic mean of precision and sensitivity. It provides a balanced measure that considers both false positives and false negatives. The F1 score is valuable when there is a need to balance the trade-off between precision and sensitivity. In medical applications, where both false positives and false negatives have consequences, the F1 score helps evaluate overall model performance.

$$F1\;Score=2\times \frac{Precision\times Sensitivity}{Precision+Sensitivity}$$

In medical applications, such as identifying heart arrhythmias, minimizing false positives and false negatives is of utmost importance. The ROC and AUC are invaluable procedures for assessing and fine-tuning the performance of classification models, ensuring that they meet the stringent requirements of accuracy and reliability necessary for medical diagnosis and decision making.

## 4. Results and Discussion

The classification model was trained in a MATLAB environment on an Ubuntu desktop with a Ryzen 9 CPU, NVIDIA RTX 2060 Super 8 GB GPU, and 32 GB of RAM. One of the well-established GoogLeNet models was considered for comparison in the study. The specific parameters for training the algorithms utilized in this study were as follows: an SGDM optimizer was used, and an initial learning rate of 0.0001 was considered for both the models. Data augmentation was also applied to the dataset for better generalization. The data augmentation operations involved randomly flipping the training images along the vertical axis and randomly scaling them up to 50% horizontally and vertically. This aided in averting the problems of overfitting.

**Figure 5:**
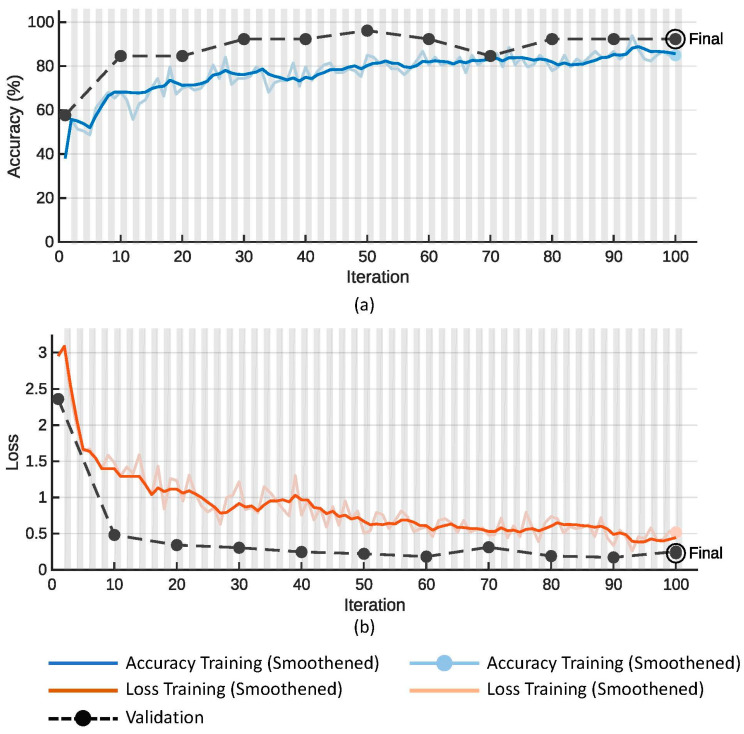
Plots of (**a**) accuracy and (**b**) loss function for the training and validation data for the proposed small CNN.

**Figure 6:**
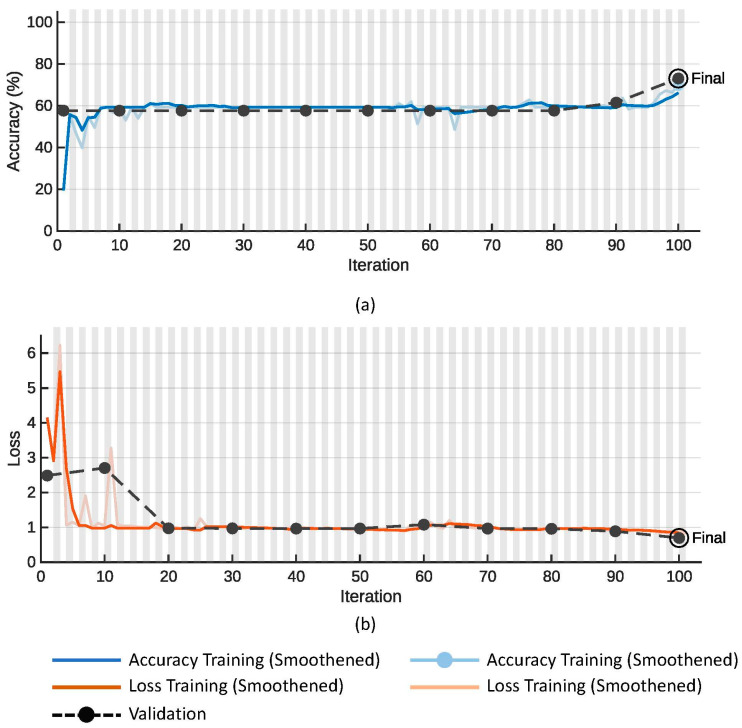
Plots of (**a**) accuracy and (**b**) loss function for the training and validation data for the GoogLeNet model.

Figure 5 shows plots of the (a) accuracy and (b) loss function on the training (colored line) and validation data (dotted line with black markers) for the proposed SmallNet CNN model. Similarly, Figure 6 shows plots of the (a) accuracy and (b) loss function on the training (colored line) and validation data (dotted line with black markers) for the GoogLeNet model.

Table 2 compares the performance of the proposed small CNN against that of GoogLeNet deep learning models across key metrics. The proposed small CNN exhibited superior overall accuracy (91.20%) and validation accuracy (92.31%) compared to GoogLeNet (78.26% and 73.08%, respectively). Additionally, the smaller model achieved these accuracies with a notably reduced training time (36 s vs. 44 s) and a significantly lower number of tunable parameters (1.5 M vs. 5.9 M) in comparison to the larger GoogLeNet models. This suggests that the proposed small CNN not only exceled in terms of accuracy but also demonstrated computational efficiency and model simplicity, emphasizing its potential as an effective and resource-efficient deep learning solution.

Table 3 provides a detailed insight into the models’ performance across different classes and presents a detailed breakdown of important performance metrics, including sensitivity, specificity, precision, and F1 score, for both the proposed small CNN and GoogLeNet models across different classes—ARR, CHF, and NSR. In terms of sensitivity, the proposed small CNN demonstrated excellent performance by correctly identifying all instances of ARR and NSR while achieving a moderate sensitivity for CHF. On the other hand, GoogLeNet achieved perfect sensitivity for ARR but struggled to identify instances of NSR and CHF. Regarding specificity, both models exceled in correctly identifying CHF cases, with the proposed small CNN achieving high specificity for NSR as well. Notably, the proposed small CNN outperformed GoogLeNet in precision and F1 score across ARR, CHF, and NSR, suggesting a more balanced performance in terms of positive predictions. The absence of precision values for CHF in “GoogLeNet” indicated potential challenges in correctly identifying this class.

**Figure 7:**
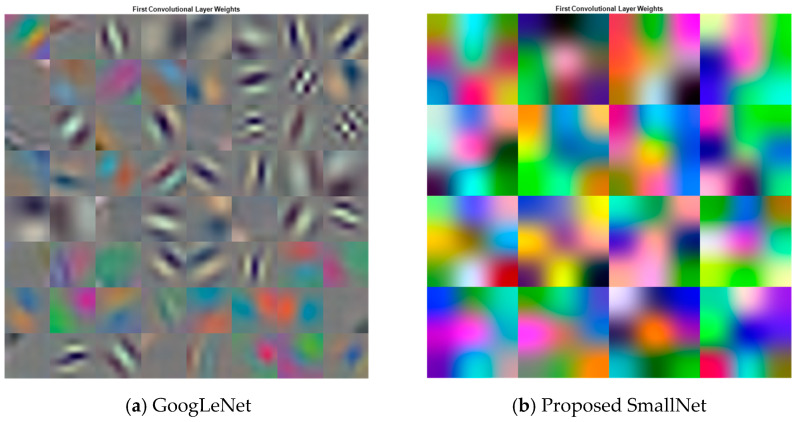
Visualization of activation patterns for first convolution layer weights in (**a**) GoogLeNet and (**b**) proposed SmallNet.

**Figure 8:**
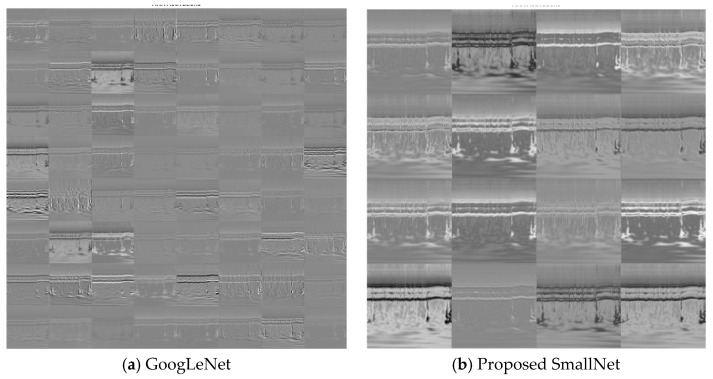
Visualization of activation patterns for first convolution layer weights in (**a**) GoogLeNet and (**b**) proposed SmallNet.

Within CNNs like GoogLeNet and the proposed SmallNet, each layer generates responses or activations in response to input images. However, not all layers are equally adept at extracting meaningful features, with early layers being particularly effective at capturing basic image features like edges and blobs. To visually demonstrate this, Figure 7a showcases the activations of GoogLeNet, while Figure 7b displays the activations for the proposed SmallNet. These visual representations, especially the filter weights of the first convolutional layer, offer insights into how each model extracts and interprets features. Notably, the figures illustrate the hierarchical nature of feature extraction, where lower layers focus on foundational patterns. Figure 8 provides an examination of convolutional layer activations for both GoogLeNet and the proposed SmallNet, focusing on an image from the ARR class. The comparison involves scrutinizing areas in the convolutional layers that become activated and contrasting them with corresponding regions in the original image. This is essential for understanding how these neural networks respond to arrhythmia-related features.

**Figure 9:**
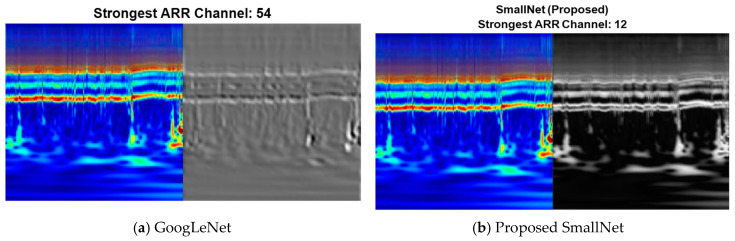
Visualization of strongest activation channels for ARR classification in proposed SmallNet and GoogLeNet.

Comparing the strongest activation channel for classification in CNNs holds significance in unraveling how these networks prioritize and respond to different features during the classification process. In the context of ARR detection, CNNs often operate on multiple channels, each corresponding to specific visual characteristics. Identifying the strongest activation channel involves pinpointing the channel that exhibits the highest level of activation or response to features relevant to the classification task. Figure 9 shows the visualization of the strongest activation channels for ARR classification in the proposed SmallNet and GoogLeNet. This visualization is essential to elucidate which visual cues or patterns contribute most significantly to the network’s decision making.

**Figure 10:**
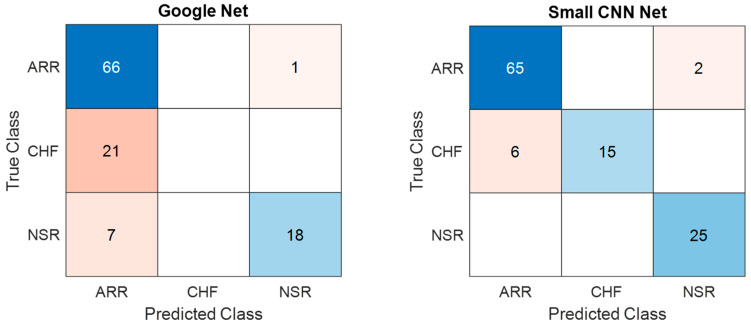
Confusion matrices for training data—comparative analysis of GoogLeNet and proposed SmallNet in cardiac arrhythmia classification.

Figure 10 presents a comparison of the confusion matrices for the training dataset obtained from both the models considered in the present study. The confusion matrices for training data obtained from GoogLeNet and the proposed SmallNet revealed nuanced classification performance. GoogLeNet demonstrated strength in accurately classifying NSR instances, with 66 ARR and 18 NSR instances correctly identified. However, it exhibited challenges in distinguishing ARR and CHF instances, misclassifying 21 CHF instances as ARR. On the other hand, the proposed SmallNet showcased proficiency in correctly classifying ARR instances, with 65 instances accurately identified. While it misclassified two ARR instances as CHF and six CHF instances as ARR, it notably outperformed GoogLeNet in CHF classification. Additionally, the proposed SmallNet exceled in NSR classification, accurately identifying all 25 instances.

**Figure 11:**
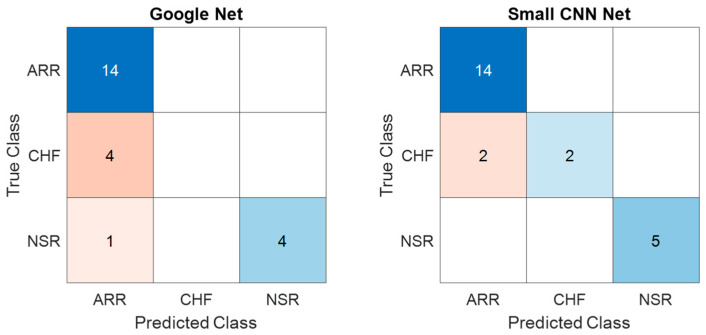
Confusion matrices for testing data—comparative analysis of GoogLeNet and proposed SmallNet in cardiac arrhythmia classification.

Similarly, the confusion matrices for the test dataset obtained from GoogLeNet and the proposed SmallNet are shown in Figure 11 and offer insights into their classification performance. The GoogLeNet confusion matrix shows that it accurately classified 14 instances of ARR and 4 instances of NSR. However, it struggled to differentiate CHF, misclassifying four CHF instances, potentially indicating challenges in distinguishing between CHF and other classes. The proposed SmallNet, on the other hand, demonstrated proficiency in ARR classification, with 14 instances accurately identified. It faced challenges in CHF classification, misclassifying two instances as ARR and two instances as NSR. Despite these challenges, it exceled in correctly classifying NSR instances, with five accurately identified.

**Figure 12:**
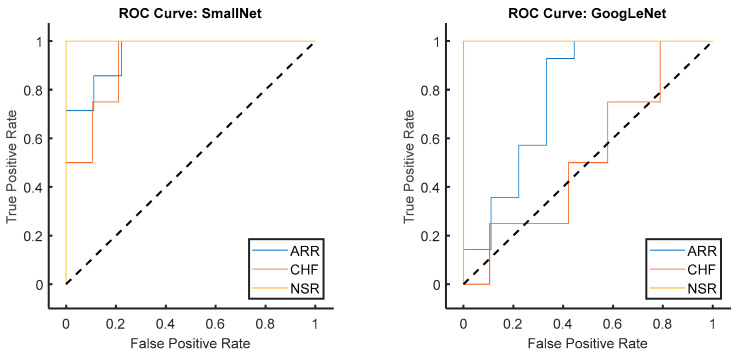
Comparison of ROC curves for the proposed small CNN and GoogLeNet.

Figure 12 shows a comparison of the ROC curves for both models, and the respective values for each class are displayed in Table 4. From Figure 11 and Table 4, it can be concluded that the provided class-wise AUC values for the proposed small CNN and GoogLeNet shed light on their performance in distinguishing between different cardiac classes. In terms of ARR detection, the proposed small CNN outperformed GoogLeNet, with an AUC value of 0.9524 compared to 0.7778, indicating a superior ability to discriminate between positive and negative instances of ARR. For CHF, the proposed small CNN again exhibited stronger discriminatory power, with an AUC value of 0.9211, while GoogLeNet lagged behind with a lower AUC of 0.5263. Remarkably, both models achieved perfect AUC values of 1 for NSR, signifying excellent performance in distinguishing NSR instances. These AUC results underscore the effectiveness of the proposed small CNN, especially in ARR and CHF detection, as reflected in the higher AUC values across these classes compared to GoogLeNet.

**Figure 13:**
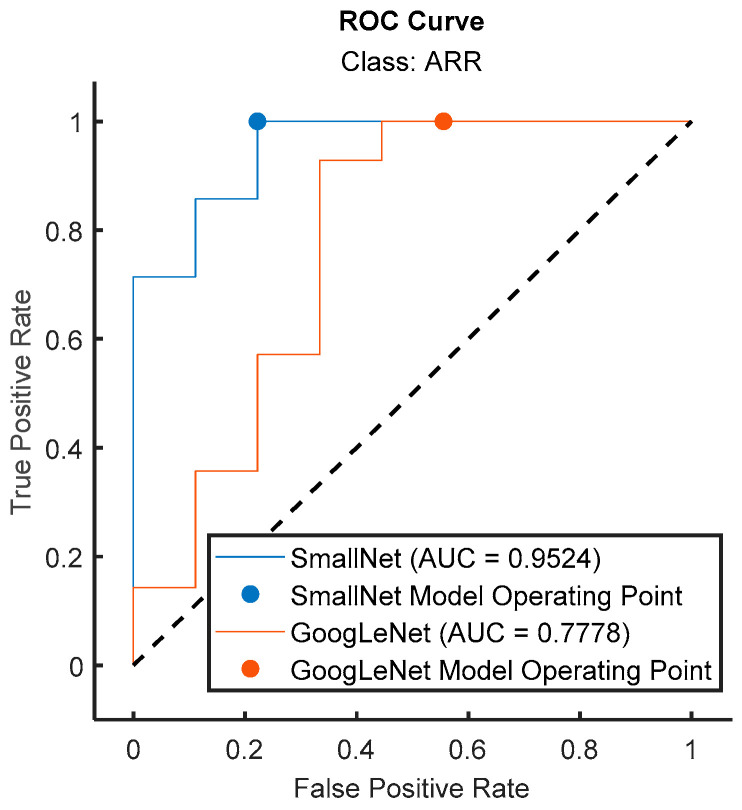
Comparison of ROC curves for the proposed small CNN and GoogLeNet for ARR class.

In Figure 13, the ROC curves for the proposed small CNN and GoogLeNet are visually compared specifically for the ARR class. The ROC curve illustrates the trade-off between the true-positive rate and false-positive rate across different classification thresholds. The accompanying AUC values provide a quantitative measure of the models’ discriminative performance. Notably, the ROC curve for the proposed small CNN showcased a superior AUC of 0.9524 for ARR detection, indicating a high level of accuracy in distinguishing between positive and negative instances of arrhythmia. In contrast, GoogLeNet exhibited a lower AUC value of 0.7778 for the same class, suggesting a less effective performance in ARR detection. This visual and numerical evidence confirms the enhanced performance offered by the proposed small CNN in accurately identifying instances of arrhythmia, reinforcing its potential as a robust model for cardiac classification tasks.

**Figure 14:**
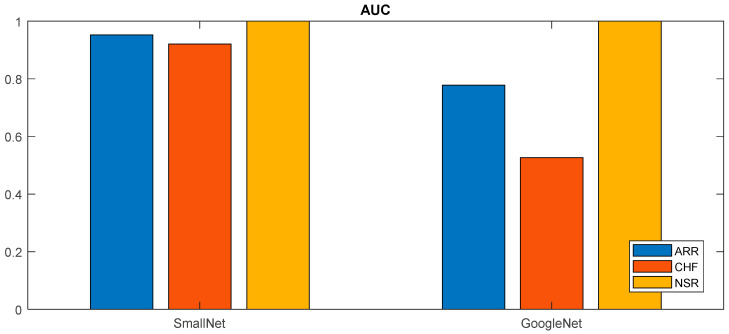
Comparative analysis of AUC values for proposed small CNN and GoogLeNet across cardiac classes.

In Figure 14, a bar graph succinctly illustrates the AUC values for the proposed small CNN and GoogLeNet across different cardiac classes. The graph provides a visual representation of the discriminative performance of each model for ARR, CHF, and NSR. Notably, the proposed small CNN consistently exhibited higher AUC values for all classes—0.9524 for ARR, 0.9211 for CHF, and a perfect AUC of 1 for NSR. This graphical representation reinforces the quantitative findings, emphasizing the proposed small CNN’s superior classification performance across the diverse range of cardiac conditions compared to GoogLeNet.

**Figure 15:**
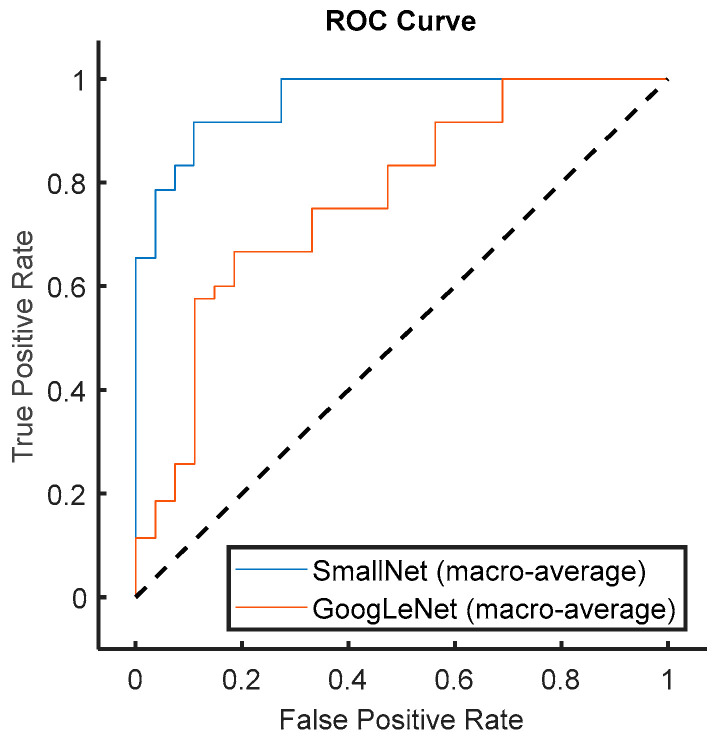
Comparison of marco-average ROC curves for the proposed small CNN and GoogLeNet.

In Figure 15, a comprehensive comparison of the average ROC curves for all classes is presented for both the proposed small CNN and GoogLeNet. The ROC curves provide a holistic view of the models’ classification performance across various thresholds. Importantly, the figure highlights that the proposed small CNN consistently outperformed GoogLeNet in terms of AUC (area under the ROC curve), indicating superior discriminative power across all classes. The average AUC value serves as a robust metric for overall classification performance, and the visually evident higher AUC for the proposed small CNN substantiates its effectiveness in accurately distinguishing between different cardiac classes. This result underscores the model’s potential as a reliable choice for a broad spectrum of cardiac classification tasks, further emphasizing its comprehensive and superior performance across diverse classes compared to GoogLeNet.

## 5. Conclusions

In conclusion, the present work provided a comprehensive exploration of deep-learning-based methods for arrhythmia identification using ECG signals. The study showcased the critical role that deep learning techniques play in the healthcare sector, particularly in the early detection and treatment of arrhythmia. By comparing the performance of a proposed small CNN model with established architectures like GoogLeNet, it was demonstrated that simplicity and efficiency need not compromise accuracy. The small CNN model exhibited remarkable accuracy in detecting arrhythmia, underscoring its potential as an effective tool for automated arrhythmia detection. This research not only advances our understanding of arrhythmia detection but also underscores the broader potential of deep learning in healthcare. Early and accurate arrhythmia detection is crucial for patient outcomes and healthcare efficiency. As technology continues to advance, deep-learning-based approaches like that presented here offer a promising path toward improving patient care and reducing the global burden of cardiovascular diseases.

## Figures and Tables

**Table 1:** Comparative overview of arrhythmia databases.

Attribute	MIT-BIH ARR Database	MIT-BIH NSR Database	BIDMC CHF Database
Type of Database	Arrhythmia (ARR)	Normal sinus rhythm (NSR)	Congestive heart failure (CHF)
Source	MIT-BIH	MIT-BIH	Beth Israel Deaconess Medical Center (BIDMC)
Data Collection Method	Holter monitoring, ECG signals	ECG signals	Clinical records, ECG signals
Number of Subjects	96 records	36 records	30 records
Recorded Parameters	ECG signals	ECG signals	Clinical parameters, ECG signals
Annotation	Annotations for arrhythmias	Annotations for normal sinus rhythm	Annotations for heart failure events, clinical annotations
Location	MIT, Massachusetts, USA	MIT, Massachusetts, USA	Boston, Massachusetts, USA
Technical Specifications	Holter monitors, ECG devices with various sampling rates and resolutions	ECG devices with various sampling rates and resolutions	ECG devices, clinical monitoring equipment with varying specifications
Sampling Rate	Typically, 360 Hz	Typically, 128 Hz	Typically, 125 Hz
Purpose	Arrhythmia research	Normal sinus rhythm research	Heart failure research and clinical studies
Challenges	Presence of arrhythmias	Limited arrhythmias, focus on normal cases	Complex clinical data, diverse conditions
Use Cases	Arrhythmia detection algorithms, cardiac research	Normal sinus rhythm analysis, baseline for comparison	Heart failure prediction, clinical studies

**Table 2:** The accuracy obtained for the two deep learning models.

Metric	Proposed Small CNN	GoogLeNet
Overall accuracy	91.20%	78.26%
Validation accuracy	92.31%	73.08%
Training time	36 s	44 s
No. of tunable parameters	1.5 M	5.9 M

**Table 3:** Class-wise evaluation metrics for proposed small CNN and GoogLeNet.

Metric	Proposed Small CNN			GoogLeNet		
	ARR	CHF	NSR	ARR	CHF	NSR
Sensitivity	1	0.5	1	1	0	0.8
Specificity	0.778	1	1	0.444	1	1
Precession	0.875	1	1	0.737	-	1
F1 score	0.933	0.667	1	0.848	-	0.889

**Table 4:** Class-wise AUC values obtained for the two models.

AUC Value	Proposed Small CNN	GoogLeNet
ARR	0.9524	0.7778
CHF	0.9211	0.5263
NSR	1	1

## Data Availability

The data presented in this study are openly available in PhysioNet repository. The three datasets (MIT-BIH ARR, MIT-BIH NSR, and BIDMC-CHF) used in the present study are available at https://doi.org/10.13026/C2F305, https://doi.org/10.13026/C2NK5R and https://doi.org/10.13026/C29G60 respectively.
